# Antimicrobial Metabolites from the Endophytic Fungus *Pichia guilliermondii* Isolated from *Paris polyphylla* var. *yunnanensis*

**DOI:** 10.3390/molecules15117961

**Published:** 2010-11-05

**Authors:** Jianglin Zhao, Yan Mou, Tijiang Shan, Yan Li, Ligang Zhou, Mingan Wang, Jingguo Wang

**Affiliations:** 1 College of Agronomy and Biotechnology, China Agricultural University, Beijing 100193, China; 2 College of Science, China Agricultural University, Beijing 100193, China; 3 College of Resources and Environmental Sciences, China Agricultural University, Beijing 100193, China

**Keywords:** endophytic fungus, *Pichia guilliermondii*, *Paris polyphylla* var. *yunnanensis*, steroids, helvolic acid, antimicrobial activity

## Abstract

Three steroids and one nordammarane triterpenoid were isolated for the first time from the endophytic fungus *Pichia guilliermondii* Ppf9 derived from the medicinal plant *Paris polyphylla* var. *yunnanensis*. By means of physicochemical and spectrometric analysis, they were identified as ergosta-5,7,22-trienol (**1**), 5α,8α-epidioxyergosta-6,22-dien-3β-ol (**2**), ergosta-7,22-dien-3β,5α,6β-triol (**3**), and helvolic acid (**4**). Both micro-dilution-colorimetric and spore germination assays were employed to evaluate their antimicrobial activity. Among them, helvolic acid (**4**) exhibited the strongest antibacterial activity against all test bacteria, with MIC values ranging from 1.56 µg/mL to 50 µg/mL, and IC_50_ values from 0.98 µg/mL to 33.19 µg/mL. It also showed strong inhibitory activity on the spore germination of *Magnaporthe oryzae* with an IC_50_ value of 7.20 µg/mL. Among the three steroids, 5α,8α-epidioxyergosta-6,22-dien-3β-ol (**2**) exhibited relatively strong antimicrobial activity. The results suggest that the endophytic fungus *Pichia guillermondii* Ppf9 could be a candidate for producing helvolic acid, and the metabolites from this fungus could be potentially developed as antimicrobial agents in the future.

## 1. Introduction

Plant endophytic fungi are fungal microorganisms which spend all or part of their lifecycle inter- and/or intra-cellularly colonizing healthy tissues of their host plants, typically causing no apparent disease symptoms [1,2]. They are an important and novel source of natural bioactive compounds with great potential applications in agriculture, medicine and the food industry [3,4,5,6]. Since the "gold" bioactive compound paclitaxel was successfully discovered in the endophytic fungus *Taxomyces andreanae* in 1993, many scientists have increased their interest in studying fungal endophytes as potential producers of novel and bioactive compounds, and over the past two decades, many valuable bioactive compounds with antimicrobial, insecticidal, cytotoxic and anticancer activities have been successfully discovered in endophytic fungi. These bioactive compounds could be mainly classified as alkaloids, terpenoids, steroids, quinones, isocoumarins, lignans, phenylpropanoids, phenols and lactones [7,8,9].

*Paris polyphylla* var. *yunnanensis* (Franch) Hand.-Mazz. (Trilliaceae), a perennial herb found mainly distributed in the Provinces of Yunnan, Sichuan and Guizhou in southwest China, has been used as an important and traditional Chinese medicine (TCM) for treatment of injuries from falls, fractures, contusions, bleeding and immunity adjustment [10,11]. Our previous investigations showed that a broad diversity of endophytic fungi existed in *P. polyphylla* var. *yunnanensis*, and the crude extracts of some endophytic fungal isolates exhibited pronounced antimicrobial activity [12,13,14]. The purpose of this study was to determine the antimicrobial components produced by the endophytic fungus *Pichia guilliermondii* Ppf9 from *P. polyphylla* var. *yunnanensis* based on previous studies [13,14], as well as to evaluate the antimicrobial activity of these compounds for their potential application as antimicrobial agents.

## 2. Results and Discussion

### 2.1. Isolation and identification

**Figure 1:**
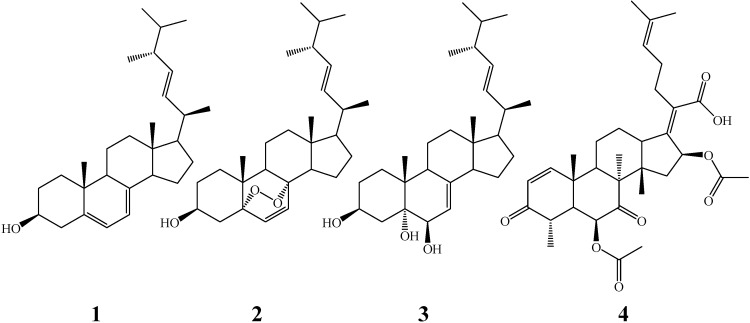
Chemical structures of the compounds ergosta-5,7,22-trienol (**1**), 5α,8α-epidi-oxyergosta-6,22-dien-3β-ol (**2**), ergosta-7,22-dien-3β,5α,6β-triol (**3**) and helvolic acid (**4**).

By bioassay-guided fractionation, four compounds were obtained from the crude extract of the endophytic fungus *Pichia guilliermondii* Ppf9 of *P. polyphylla* var. *yunnanensis* for the first time. After comparing their physicochemical and spectral data with those found in the literature [15,16,17,18], they were identified as ergosta-5,7,22-trienol (**1**), 5α,8α-epidioxyergosta-6,22-dien-3β-ol (**2**), ergosta-7,22-dien-3β,5α,6β-triol (**3**), and helvolic acid (**4**), which structures were shown in Figure 1. 5α,8α-Epidioxyergosta-6,22-dien-3β-ol (**2**) had been isolated from the endophytic fungus *Fusarium* sp. Ppf4 in our previous study [19], and it has also been obtained from other endophytic fungi, such as *Aspergillus fumigatus* CY018 and *Aspergillus* sp. CY725 derived from the leaves of *Cynodon dactylon* [20,25], and *Colletotrichum* sp. from *Artemisia annua* [21]. Helvolic acid (**4**), a nordammarane triterpenoid antibiotic, has been successfully obtained from other fungi such as the entomopathogenic fungus *Metarhizium anisopliae* [18], the rice fungal pathogen *Sarocladium oryzae* [22], the marine-derived fungus *Aspergillus sydowi* [23], endophytic *Alternaria* sp. FL25 from *Ficus carica* [24], and endophytic *Aspergillus* sp. CY725 from *Cynodon dactylon* [25]. This study revealed that there was a diversity of metabolites in *P. guilliermondii* except in the volatile oil, as previously reported [14].

### 2.2. Antimicrobial activity

The antimicrobial activities of these compounds were further evaluated by micro-dilution-colorimetric and spore germination assays with the results shown in Table 1. The antimicrobial activity assay indicated that helvolic acid (**4**) should be the main antimicrobial component in endophytic fungus *P. guilliermondii* Ppf9, as this compound exhibited the strongest antibacterial activity on *A. tumefaciens*, *E. coli*, *P. lachrymans*, *R. solanacearum*, *X. vesicatoria*, *B. subtilis*, *S. aureus* and *S. haemolyticus*, with minimum inhibitory concentration (MIC) values of 1.56, 3.13, 3.13, 1.56, 1.56, 3.13, 50 and 6.25 µg/mL, respectively. Correspondingly, the median inhibitory concentration (IC_50_) values were 0.95, 2.04, 1.45, 0.94, 0.98, 2.11, 33.19 and 3.25 µg/mL, respectively. Its antibacterial activity was close to or a little stronger than that of streptomycin sulfate (the positive control). Helvolic acid also showed strong inhibitory activity on the spore germination of *M. oryzae*, with an IC_50_ value of 7.20 µg/mL. Helvolic acid (**4**) has been screened to show strong antimicrobial activity against a wide range of microorganisms including bacteria (*i.e.* *Bacillus sublitis*, *Clavibacter michiganensis*, *Escherichia coli*, *Helicobacter pylori*, *Micrococcus lysoleikticus*, *Pseudomonas aeruginosa*, *Sarcina lutea*, *Staphylococcus aureus*, *Streptococcus lactis*) and fungi (i.e. *Alternaria brassicae*, *Botrytis cinerea*, *Candida albicans*, *Colletotirchum gloeosporioides*, *Fusarium graminearum*, *Phytophthora capsici* and *Valsa mali*) [23,24,25].

Among the three isolated steroids 5α,8α-epidioxyergosta-6,22-dien-3β-ol (**2**) exhibited relatively strong antimicrobial activity and we can speculate that the peroxide bridge between C-5 and C-8 positions may be crucial for the antimicrobial activity. In previous reports, 5α,8α-epidioxyergosta-6,22-dien-3β-ol (**2**) has been shown to have obvious antibacterial activity against *Agrobacterium tumefaciens*, *Bacillus subtilus*, *Escherichia coli*, *Helicobacter pylori*, *Pseudononas* sp., *Sarcina lutea*, *Staphylococcus aureus*, *Staphylococcus haemolyticus* and *Xanthomonas vesicatoria*, as well as antifungal activity on *Aspergillus niger*, *Candida albicans* and *Magnaporthe oryzae* [19,21,25]. Otherwise, other bioactivities such as anti-inflammatory [26], anticancer [26], cyclooxygenase inhibitory [27], and antioxidant [28] properties of this compound were also shown be significant in the corresponding screens. Overall our results were in agreement with these previous reports.

**Table 1:** Antimicrobial activity of the compounds **1**-**4** obtained from *P. guilliermondii* Ppf9.

Test Microorganism	MIC and IC_50_ of the compound (μg/mL)									
	1		2		3		4		Positive control	
	MIC	IC_50_	MIC	IC_50_	MIC	IC_50_	MIC	IC_50_	MIC	IC_50_
*A. tumefaciens*	100	55.65±0.68	50	30.79±0.41	200	111.52±1.65	1.56	0.95 ± 0.08	6.25	3.83 ± 0.05
*E. coli*	200	135.10 ± 1.32	150	85.90 ± 0.53	>200	nd	3.13	2.04 ± 0.12	25	7.55 ± 0.18
*P. lachrymans*	100	65.24 ± 0.56	50	33.96 ± 0.25	150	85.03 ± 0.72	3.13	1.45 ± 0.06	12.5	6.82 ± 0.10
*R. solanacearum*	150	93.05 ± 0.71	100	67.18 ± 0.31	150	95.98 ± 0.86	1.56	0.94 ± 0.02	12.5	6.75 ± 0.06
*X. vesicatoria*	150	105.89 ± 1.02	100	70.75 ± 0.46	150	90.75 ± 1.32	1.56	0.98 ± 0.04	12.5	5.72 ± 0.12
*B. subtilis*	150	93.89 ± 1.15	100	62.99 ± 0.18	200	128.96 ± 0.71	3.13	2.11 ± 0.10	50	27.35 ± 1.06
*S. aureus*	>200	nd	>200	nd	>200	nd	50	33.19 ± 0.52	100	65.98 ± 1.32
*S. haemolyticus*	200	145.36 ± 0.67	150	109.72 ± 0.85	>200	nd	6.25	3.25 ± 0.04	50	31.94 ± 1.18
*M. oryzae*	nd	126.91 ± 1.22	nd	81.95 ± 0.62	nd	110.87 ± 0.65	nd	7.20 ± 0.08	nd	2.86 ± 0.16

Note: MIC, minimum inhibitory concentration. IC_50_, median inhibitory concentration. Positive controls for bacteria and fungus *M. oryzae* were streptomycin sulfate and carbendazim, respectively. The 'nd' means not detected. Mean ± standard deviation of three independent experiments (three replicates for each treatment).

## 3. Experimental

### 3.1. General

Melting points of these compounds were measured on an XT4-100B microscopic melting-point apparatus (Tianjin Tianguang Optical Instruments Company, China) and are uncorrected. NMR spectra were recorded on a Bruker Avance DRX-500 spectrometer (^1^H at 500 MHz and ^13^C at 125 MHz) using tetramethylsilane (TMS) as the internal standard, and chemical shifts were recorded as *δ* values. ESI-MS spectra were recorded on a Bruker Esquire 6000 LC/MS spectrometer. Both silica gel (200-300 mesh) for column chromatography (CC) and silica gel GF_254_ (10-20 mm) for thin layer chromatography (TLC) were acquired from the Qingdao Marine Chemical Company, China. The Sephadex LH-20 and silica gel RP-18 were purchased from Pharmacia Biotech, Sweden. A microplate spectrophotometer (PowerWave HT, BioTek Instruments, USA) was employed to measure the light absorption value. Carbendazim and streptomycin sulfate were purchased from Sigma-Aldrich (USA). 3-(4,5-Dimethylthiazol-2-yl)-2,5-dephenyl tetrazolium bromide (MTT) was purchased from Amresco (USA). All other chemicals and reagents were of analytical grade.

### 3.2. Fungal material

The endophytic fungal isolate Ppf9 was isolated from healthy rhizomes of the medicinal plant *P. polyphylla* var. *yunnanensis*, and identified through its morphological characteristics and internal transcribed spacer (ITS) rRNA gene sequence analysis (GenBank accession number EF 495244), which gave a 99.0% sequence similarity to *Pichia guilliermondii* (DQ 663478). The living culture has been deposited in the China General Microbiological Culture Collection Center (CGMCC) under the number CGMCC 2475. It is also stored on PDA slants at 4 ºC and in 40% glycerol at -70 ºC in the Herbarium of the College of Agronomy and Biotechnology, China Agricultural University. Both the crude extract and volatile oil of this endophytic fungus were screened to show obvious antimicrobial activity in our previous studies [13,14].

### 3.3. Fermentation, extraction and isolation

The endophytic fungus *P. guilliermondii* Ppf9 was cultured on PDA (potato 200 g/L, dextrose 20 g/L, and agar 20 g/L) medium in Petri dishes at 25 ºC for 5 days. Then, two to three plugs of agar medium (0.5 × 0.5 cm) with fungal cultures were inoculated in each 1000-mL Erlenmeyer flask containing 300 mL potato dextrose broth (PDB) medium, and incubated on a rotary shaker at 150 rpm and 25 ºC for 7 days. Afterwards, a total of 50 L fermentation broth was harvested. The fungal cells separated from the culture filtrate by filtration was dried and powdered, and extracted for five times with acetone. The culture filtrate was concentrated and extracted with *n*-butanol five times. The acetone and *n*-butanol extractions were concentrated under vacuum at 50 ºC on a rotary evaporator to obtain the crude extracts. As the TLC and TLC-bioautographic-assays were similar to each other, both the acetone and *n*-butanol extractions were combined, and a total of 65.0 g crude extract was obtained. 

The crude extract was firstly subjected to a column chromatagraphy (CC) over silica gel (200-300 mesh) eluted with CHCl_3_-MeOH (100:15, v/v) to obtain nine fractions (Frs. 1-9). The fractions were monitored by TLC, and similar fractions were combined to afford four composite fractions: FA (Fr.1-4), FB (Fr.5-6), FC (Fr.7-8) and FD (Fr.9). According to the TLC-bioautography-assay of these four fractions, FA was screened to show strong antimicrobial activity, and it was selected for further fractionation on a silica gel column eluted with cyclohexane-acetone (from 1:0 to 0:1, v/v) to give five subfractions (FA-1 to 5). FA-1 (0.12 g) was purified by Sephadex LH-20 (CHCl_3_-MeOH = 1:1, v/v) and recrystalization to afford **1** (35.0 mg). FA-2 (0.16 g) was refractionated by CC on silica gel eluted with cyclohexane-acetone (100:20, v/v), and was further purified by Sephadex LH-20 (CHCl_3_-MeOH = 1:1, v/v) and RP-18 column (H_2_O-MeOH = 1:9, v/v) to yield **2** (13.6 mg). FA-4 (0.10 g) was subjected to CC over silica gel eluted with cyclohexane-acetone (100:50, v/v), and was further purified over Sephadex LH-20 (CHCl_3_-MeOH = 1:1, v/v) to afford **4** (15.3 mg). FA-5 (0.08 g) was purified by Sephadex LH-20 (CHCl_3_-MeOH = 1:1, v/v) and recrystallization to afford **3** (10.5 mg). The physicochemical and spectrometric data of these compounds are given as follows.

*Ergosta-5,7,22-trienol* (**1**). Colourless needles (CHCl_3_), m.p. 168-170 ºC; ESI-MS m/z 397 [M+H]^+^; ^1^H-NMR (CDCl_3_) *δ* (ppm), 5.57 (1H, dd, *J* = 6.6, 2.5 Hz, H-6), 5.36 (1H, m, H-7), 5.24 (1H, dd, *J =* 15.9, 6.6 Hz, H-23), 5.17 (1H, dd, *J* = 15.9, 6.6 Hz, H-22), 3.60 (1H, m, H-3), 1.02 (3H, d, *J =* 6.6 Hz), 0.95 (3H, s), 0.92 (3H, d, *J =* 6.8 Hz), 0.84 (3H, d, *J =* 6.6 Hz), 0.82 (3H, d, *J =* 6.7 Hz), 0.63 (3H, s); ^13^C-NMR (CDCl_3_) *δ* (ppm), 38.5 (C-1), 32.0 (C-2), 70.3 (C-3), 40.8 (C-4), 139.8 (C-5), 119.6 (C-6), 116.3 (C-7), 141.4 (C-8), 46.3 (C-9), 37.1 (C-10), 21.0 (C-11), 39.5 (C-12), 43.0 (C-13), 54.5 (C-14), 28.2 (C-15), 23.0 (C-16), 56.0 (C-17), 12.0 (C-18), 16.3 (C-19), 40.0 (C-20), 21.1 (C-21), 132.0 (C-22), 135.6 (C-23), 42.8 (C-24), 33.1 (C-25), 19.6 (C-26), 20.0 (C-27), 17.6 (C-28). It was further identified by comparison with the authentic sample by TLC. The structure was confirmed by comparison with literature data [15].

*5α,8α-Epidioxyergosta-6,22-dien-3β-ol* (**2**). Colourless needles (CHCl_3_); m.p. 176-178 ºC; ESI-MS m/z 429 [M+H]^+^; ^1^H-NMR (CDCl_3_) *δ* (ppm), 6.52 (1H, d, *J =* 8.5 Hz, H-7), 6.26 (1H, d, *J =* 8.5 Hz, H-6), 5.22 (1H, dd, *J =* 15.3, 7.8 Hz, H-22), 5.18 (1H, dd, *J =* 15.3, 7.8 Hz, H-23), 3.97 (1H, m, H-3), 1.01 (3H, d, *J =* 6.6 Hz, H-21), 0.92 (3H, d, *J =* 6.8 Hz, H-28), 0.90 (3H, s, H-19), 0.85 (3H, d, *J =* 6.8 Hz, H-26), 0.84 (3H, s, H-18), 0.81 (3H, d, *J =* 6.8 Hz, H-27); ^13^C-NMR (CDCl_3_) *δ* (ppm), 34.9 (C-1), 30.3 (C-2), 66.7 (C-3), 37.2 (C-4), 82.4 (C-5), 135.6 (C-6), 130.9 (C-7), 79.7 (C-8), 51.2 (C-9), 37.1 (C-10), 23.6 (C-11), 39.5 (C-12), 44.8 (C-13), 51.9 (C-14), 20.9 (C-15), 28.9 (C-16), 56.4 (C-17), 13.1 (C-18), 18.4 (C-19), 40.0 (C-20), 21.1 (C-21), 135.4 (C-22), 132.4 (C-23), 42.9 (C-24), 33.3 (C-25), 20.2 (C-26), 19.9 (C-27), 17.8 (C-28). The structure was confirmed by comparison with literature data [16].

*Ergosta-7,22-dien-3β,5α,6β-triol* (**3**). Colourless needles (MeOH); m.p. 226-228 ºC; ESI-MS m/z 431 [M+H]^+^; ^1^H-NMR (C_5_D_5_N) *δ* (ppm), 5.74 (1H, dd, *J =* 4.8, 2.4 Hz, H-7), 5.25 (1H, dd, *J =* 15.5, 7.3 Hz, H-23), 5.18 (1H, dd, *J =* 15.5, 7.8 Hz, H-22), 4.83 (1H, m, H-3), 4.31 (1H, brs, H-6), 1.07 (3H, d, *J =* 6.5 Hz, H-21), 0.96 (3H, d, *J =* 6.8 Hz, H-28), 0.93 (3H, s, H-19), 0.87 (3H, d, *J =* 6.6 Hz, H-27), 0.86 (3H, d, *J =* 6.6 Hz, H-26), 0.67 (3H, s, H-18); ^13^C-NMR (C_5_D_5_N) *δ* (ppm), 32.6 (C-1), 33.8 (C-2), 67.6 (C-3), 41.9 (C-4), 76.2 (C-5), 74.3 (C-6), 120.4 (C-7), 141.6 (C-8), 43.8 (C-9), 38.1 (C-10), 22.4 (C-11), 40.0 (C-12), 43.8 (C-13), 55.3 (C-14), 23.5 (C-15), 28.4 (C-16), 56.3 (C-17), 12.5 (C-18), 18.8 (C-19), 40.8 (C-20), 20.1 (C-21), 136.2 (C-22), 132.2 (C-23), 43.1 (C-24), 33.4 (C-25), 20.7 (C-26), 19.9 (C-27), 17.9 (C-28). The structure was confirmed by comparison with literature data [16].

*Helvolic acid* (**4**). Colourless needles (MeOH); m.p. 214-216 ºC; ESI-MS m/z 591 [M+Na]^+^; ^1^H-NMR (CDCl_3_) *δ* (ppm), 7.31 (1H, d, *J =* 10.0 Hz, H-1), 5.88 (1H, d, *J =* 8.5 Hz, H-16), 5.87 (1H, d, *J =* 10.0 Hz, H-2), 5.24 (1H, s, H-6), 5.11 (1H, t, *J =* 7.0 Hz, H-24), 2.78 (1H, dq, *J =* 12.5, 7.0 Hz, H-4), 2.62 (1H, dd, *J =* 13.5, 2.5 Hz, H-9), 2.57 (1H, brd, *J =* 11.0, H-13), 2.48 (2H, m, H-22), 2.42 (1H, m, H-12), 2.28 (1H, d, *J =* 12.5 Hz, H-5), 2.23 (1H, dd, *J =* 15.0, 8.5 Hz, H-15), 2.14 (1H, m, H-23), 2.11 (3H, s, 6-OCOCH_3_), 2.09 (1H, m, H-23), 1.98 (1H, m, H-11), 1.95 (3H, s, 16-OCOCH_3_), 1.92 (1H, d, *J =* 15.0 Hz, H-15), 1.81 (1H, dd, *J =* 13.0, 3.5 Hz, H-12), 1.69 (3H, s, H-27), 1.61 (3H, s, H-26), 1.57 (1H, m, H-11), 1.45 (3H, s, H-19), 1.28 (3H, d, *J =* 7.0 Hz, H-28), 1.18 (3H, s, H-29), 0.93 (3H, s, H-18); ^13^C-NMR (CDCl_3_) *δ* (ppm), 157.2 (C-1), 127.8 (C-2), 201.3 (C-3), 40.4 (C-4), 47.2 (C-5), 73.8 (C-6), 168.9 (6-OCOCH_3_), 20.7 (6-OCOCH_3_), 208.7 (C-7), 52.6 (C-8), 41.7 (C-9), 38.1 (C-10), 23.9 (C-11), 25.9 (C-12), 49.4 (C-13), 46.6 (C-14), 40.6 (C-15), 73.4 (C-16), 170.1 (16-OCOCH_3_), 20.5 (16-OCOCH_3_), 147.8 (C-17), 17.9 (C-18), 27.5 (19), 130.2 (C-20), 173.7 (C-21), 28.6 (C-22), 28.3 (C-23), 122.7 (C-24), 132.9 (C-25), 17.9 (C-26), 25.7 (C-27), 13.1 (C-28), 18.3 (C-29). The structure was confirmed by comparison with literature data [17,18].

### 3.4. Antimicrobial activity

#### 3.4.1. Antibacterial activity assay

Five Gram-negative (*Agrobacterium tumefaciens* ATCC 11158, *Escherichia coli* ATCC 29425, *Pseudomonas lachrymans* ATCC 11921, *Ralstonia solanacearum* ATCC 11696 and *Xanthomonas vesicatoria* ATCC 11633) and three Gram-positive (*Bacillus subtilis* ATCC 11562, *Staphylococcus aureus* ATCC 6538 and *Staphylococcus haemolyticus* ATCC 29970) bacteria were selected for antibacterial activity assay. They were grown in liquid Luria-Bertani (LB) medium (yeast extract 5 g/L, peptone 10 g/L, NaCl 5 g/L, pH 7.0) overnight at 28 ºC, and the diluted bacterial suspension (1 × 10^6^ cfu/mL) was ready for detection. A modified micro-dilution-colorimetric assay by using the chromogenic reagent 3-(4,5-dimethylthiazol-2-yl)-2,5-dephenyl tetrazolium bromide (MTT) was used to detect the antibacterial activity of these compounds according to our previous report [29]. In brief, the test compound was dissolved in acetone at an initial concentration of 4.0 mg/mL. Then it was diluted with 30% acetone to obtain concentrations ranging from 3.9 μg/mL to 2.0 × 10^3^ μg/mL. Test sample solutions (10 µL) and prepared bacterial suspension (90 µL) containing 1 × 10^6^ cfu/mL were added into each well of the 96-well microplate. Each well of the negative control contained 90 µL of the inoculum (1 × 10^6^ cfu/mL) and 10 µL of 30% acetone. Streptomycin sulfate was used as the positive control. After the plates were agitated to mix the contents of the wells using a plate shaker and incubated in the dark at 28 ºC for 24 h, 10 µL of MTT (5 mg/mL in 0.2 mol/L, pH 7.2, phosphate-buffered saline, PBS) was added into each well, and the plates were incubated for another 4 h. The minimum inhibitory concentration (MIC) value was defined as the lowest sample concentration that inhibited visible growth of the test bacterium, as indicated by the MTT staining. Only living microorganisms can convert MTT to formazan, and a blue color appeared in the well [30].

To further determine the median inhibitory concentration (IC_50_) value of each sample, the above MTT stained suspension was centrifuged at 1,500 *g* for 20 min. Then the supernatant was aspirated, 150 µL of dimethyl sulfoxide (DMSO) was added into each well, and the colored formazan products were extracted for 30 min. After complete extraction, the plate was centrifuged at 1,500 *g* for another 20 min, and then 100 µL of the supernatant in each well was transferred to a corresponding well of another 96-well microplate to measure their light absorption values at wavelength 510 nm using a microplate spectrophotometer. The percentage of the bacterial growth inhibition was determined as: Bacterial growth inhibition = [(*A*_c_-*A*_t_)/*A*_c_] × 100%, where *A*_c_ was an average of three replicates of light absorption values at wavelength 510 nm of the negative controls, and *A*_t_ was the average of three replicates of light absorption values of the samples. The IC_50_ value was calculated using the linear relation between the inhibitory probability and concentration logarithm according to the method of Sakuma [31].

#### 3.4.2. Antifungal activity assay

Rice blast fungus, *Magnaporthe oryzae* (P131), was kindly provided by Prof. Youliang Peng at the Department of Plant Pathology, China Agricultural University. It was maintained on oatmeal-tomato agar (oatmeal 30 g/L, tomato juice 150 mL/L, and agar 20 g/L) at 25 ºC. A spore germination assay was employed to detect the antifungal activity of these compounds. Briefly, the spores were prepared from 7-day-old cultures of *M. oryzae*, according to our previous reports [32,33]. The test compound-acetone solution (25 µL) was mixed with an equivalent volume of fungal spore suspension containing 2 × 10^6^ spores/mL. The mixture was then placed on separate concave glass slides. The final compound concentrations ranged from 1.56 µg/mL to 200 µg/mL in 5% (v/v) acetone. The negative control was 5% acetone, and the positive control was carbendazim with concentrations ranging from 0.78 µg/mL to 50 µg/mL. Three replicates were used for each treatment. Slides containing the spores were incubated in a moist chamber at 25 ºC for 7 h. Each slide was then observed under the microscope for spore germination status. About 100 spores per replicate were observed to detect spore germination according to the method by Fiori *et al*. [34]. The percentage of spore germination inhibition was determined as: Spore germination inhibition = [(*G*_c_-*G*_t_)/*G*_c_] × 100%, where *G*_c_ is an average of three replicates of germinated spore number in the negative control, and *G*_t_ is an average of three replicates of germinated numbers in the treated sets. The IC_50_ value calculation for the spore germination inhibition was the same as that for antibacterial activity assay.

## 4. Conclusions

In this study, we report for the first time the antimicrobial metabolites from the endophytic fungus *P. guilliermondii* Ppf9, isolated from *P. polyphylla* var. *yunnanensis*. By bioassay-guided fractionation, three steroids and one nordammarane triterpenoid were successfully obtained from the crude extract of *P. guilliermondii* Ppf9 endophyte cultures. The antimicrobial activity assay showed that helvolic acid (**4**), which exhibited strong, broad spectrum antimicrobial activity may be considered the main antimicrobial component of endophytic fungus Ppf9. 5α,8α-Epidioxyergosta-6,22-dien-3β-ol (**2**) displayed moderate activity against the test microorganisms. The present study provided the chemical basis for the efficacy of this endophytic fungus against microbial pathogens, as well as some basic information for the potential use of these compounds (e.g., helvolic acid and 5α,8α-epidioxyergosta-6,22-dien-3β-ol) as antimicrobial agents to control plant and animal diseases. The results suggested that the endophytic fungus *P. guillermondii* Ppf9 could be a candidate for producing helvolic acid. Our previous study revealed that the volatile oil from this fungus also had pronounced antimicrobial activity, and a total of 27 components were identified by GC-MS in the oil [14]. It is promising that the metabolites from endophyte *P. guilliermondii* Ppf9 could be potentially developed as antimicrobial agents in the future, though there are still some issues (such as the mechanisms of action of these antimicrobial compounds, the physiological and ecological roles of this fungus, and efficient strategies for increasing metabolite content and yield of the fungal culture) that need to be further clarified and resolved.
